# The CLEAR Principle: organizing data and metadata into semantically meaningful types of FAIR Digital Objects to increase their human explorability and cognitive interoperability

**DOI:** 10.1186/s13326-025-00340-7

**Published:** 2025-10-28

**Authors:** Lars Vogt

**Affiliations:** TIB Leibniz Information Centre for Science and Technology, Welfengarten 1B, 30167 Hanover, Germany

## Abstract

**Background:**

Ensuring the FAIRness (Findable, Accessible, Interoperable, Reusable) of data and metadata is an important goal in both research and industry. Knowledge graphs and ontologies have been central in achieving this goal, with interoperability of data and metadata receiving much attention. This paper argues that the emphasis on machine-actionability has overshadowed the essential need for human-actionability of data and metadata, and provides three examples that describe the lack of human-actionability within knowledge graphs.

**Results:**

The paper propagates the incorporation of cognitive interoperability as another vital layer within the European Open Science Cloud Interoperability Framework and discusses the relation between human explorability of data and metadata and their cognitive interoperability. It suggests adding the CLEAR Principle to support the cognitive interoperability and human contextual explorability of data and metadata. The subsequent sections present the concept of semantic units, elucidating their important role in attaining CLEAR. Semantic units structure a knowledge graph into identifiable and semantically meaningful subgraphs, each represented with its own resource that constitutes a FAIR Digital Object (FDO) and that instantiates a corresponding FDO class. Various categories of FDOs are distinguished. Each semantic unit can be displayed in a user interface either as a mind-map-like graph or as natural language text.

**Conclusions:**

Semantic units organize knowledge graphs into levels of representational granularity, distinct granularity trees, and diverse frames of reference. This organization supports the cognitive interoperability of data and metadata and facilitates their contextual explorability by humans. The development of innovative user interfaces enabled by FDOs that are based on semantic units would empower users to access, navigate, and explore information in CLEAR knowledge graphs with optimized efficiency.

## Introduction

Since their initial publication in 2014 [1, 2], the **FAIR Guiding Principles** for scientific data management and stewardship have gained significant recognition and are now regarded as essential across different domains of research and industry. The principles serve as a foundation for establishing policies, standards, and practices that ensure **F**indability, **A**ccessibility, **I**nteroperability, and **R**eusability (FAIR) of scientific data and metadata, benefiting both machines and humans alike. Addressing major societal challenges such as biodiversity loss and climate change [3] necessitates the collection, integration, and analysis of large volumes of data from various sources and stakeholders in a truly interdisciplinary approach, which would significantly benefit from the relevant data and metadata being FAIR [4].

The FAIR Guiding Principles provide the basis for the ongoing development of the European Open Science Cloud (EOSC) for shaping a FAIR ecosystem [5, 6]. To this end, the notion of **FAIR Digital Objects** (FDOs) has garnered attention as a central conceptual foundation within EOSC [7]. Each FDO is distinguished by a Globally Unique Persistent and Resolvable Identifier (GUPRI), adheres to common and preferably open file formats, is accompanied by further GUPRIs that provide richly documented metadata adhering to standards and established vocabularies, and includes contextual documentation that, in combination, guarantees an FDO’s reliable discovery, citation, and reuse. Some FDOs form containers for sets of finer-grained FDOs. Consequently, FDOs can exist at different levels of granularity, allowing for hierarchical structures of nested FDOs.

As atomic entities of the FAIR ecosystem, FDOs take a central role in the **EOSC Interoperability Framework** [6] by providing, together with suitable tools and controlled vocabularies such as ontologies, the necessary concepts for achieving interoperability. The overarching goal of this framework, when embedded in a FAIR ecosystem, is to establish a general standard in science and industry. This standard holds the potential to address various ongoing issues in scientific research, including the reproducibility crisis [8], and to enhance the overall trustworthiness of information as enshrined in the TRUST Principles of Transparency, Responsibility, User Focus, Sustainability, and Technology [9].

However, the realization of a FAIR ecosystem hinges on the availability of practical and reliable tools capable of technical implementation. In this context, the strategic utilization of **ontologies** and **knowledge graphs (KGs)**, complemented by a consistent application of **semantic data schemata**, emerges as a promising technological avenue [10–15]. Notably, KGs, underpinned by graph-based abstractions, are said to surpass relational or other NoSQL models in several aspects: (i) they provide an intuitive representation of relationships akin to mind-maps, which most users are familiar with; (ii) they facilitate flexible evolution of data schemata, which is particularly valuable for handling incomplete knowledge; (iii) they employ formalisms like ontologies and rules for machine-actionable knowledge representation; (iv) they facilitate graph analytics and machine learning techniques; and (v) they utilize specialized graph query languages supporting not only standard relational operators such as joins, unions, and projections, but also navigational operators for recursively searching entities along arbitrary-length paths [13, 16–21]. KGs, ontologies, and semantic data schemata have the potential to enhance transparency in data-driven decision-making processes and improve communication across various domains, including research, science, and industry.

While these semantic technologies hold great promises for enabling data and metadata interoperability and being essential cornerstones in a going FAIR strategy, they also come with their own technical, conceptual, and societal challenges. For instance, defining the boundaries of the concept of a KG remains somewhat ambiguous [13], considering that KGs encompass various technical and conceptual incarnations, such as labeled property graphs (e.g., *Neo4J*) and approaches based on the Resource Description Framework (RDF), the use of RDF-stores, and the application of Description Logics using the Web Ontology Language (OWL). Not all KGs use ontologies (or other controlled vocabularies) and pre-defined semantic data schemata, resulting in varying degrees of FAIRness and semantic interoperability within a KG and across different KGs. Moreover, there has been a predominant focus on achieving FAIRness of data and metadata for machines, potentially overlooking the importance of FAIRness for human users and software developers interacting with the KGs.

This paper addresses the human-actionability aspect of FAIRness of data and metadata and discusses in the *Problem statement* section three critical challenges of KGs that arise when focusing predominantly on machine-actionability (see Box 1 for a description of some terminological and representational conventions that were followed throughout this paper).

**Box 1:** Conventions

This paper refers to KGs as machine-actionable semantic graphs used for documenting, organizing, and representing lexical, assertional (e.g., empirical data), contingent, and universal statements, combining instance graphs and class axioms from ontology classes. This distinguishes KGs from ontologies as we understand them, with ontologies primarily containing universal statements (class axioms) and lexical statements. The discussion in this paper revolves around KGs and ontologies within the context of RDF-based triple stores, OWL, and Description Logics as a formal framework for inferencing. Labeled property graphs can be considered as an alternative to triple stores. These are considered, due to their widespread use, as technologies and logical frameworks for KGs that are supported by a broad community of users and developers and for which accepted standards exist. While acknowledging the existence of alternative technologies and frameworks, such as those supporting n-tuples syntax and more advanced logics like First-Order Logic [22, 23], it is important to note that these alternatives lack comprehensive tool support and widespread usage, thus hindering their transformation into well-supported, scalable, and easily usable KG applications. Throughout this paper, *regular underlined* text is used to indicate ontology classes, *italicsUnderlined* text when referring to properties (i.e., relations in labeled property graphs), and ID numbers are included to specify each of them. ID numbers are composed of the ontology prefix followed by a colon and a number (e.g., *isAbout* (IAO:0000136)). In cases where a term is not covered in any ontology, it is denoted by an asterisk (*), for example, the class *metric measurement statement unit*. Instances of classes are indicated using *‘regular underlined’* text, where the label denotes the class label and the ID number corresponds to the class. Furthermore, the term *resource* refers to something uniquely designated, such as a Globally Unique Persistent and Resolvable Identifier (GUPRI), about which you want to say something. It thus stands for something and represents something you want to talk about. In RDF, the *Subject* and the *Predicate* in a triple statement are always resources, while the *Object* can be either a resource or a literal. Resources can encompass properties, instances, or classes, with properties occupying the *Predicate* position in a triple, instances referring to individuals (= particulars), and classes representing universals. For the sake of clarity, resources are represented in the text and in figures using human-readable labels instead of their GUPRIs, with the implicit assumption that each resource possesses its own GUPRI.	

In *The CLEAR Principle* section, we argue that the **cognitive interoperability** of data and metadata for human users and software developers should be given due consideration, emphasizing thereby the importance of **contextual knowledge graph exploration**. We introduce the **CLEAR Principle**, which emphasizes on **C**ognitively interoperable, semantically **L**inked, contextually **E**xplorable, intuitively **A**ccessible, and human-**R**eadable and -interpretable data and metadata. The CLEAR Principle complements FAIR, CARE^1^, and TRUST by addressing the human-centric aspects of data and metadata findability, accessibility, interoperability, and reusability—something largely missing in current frameworks. Due to the potential of ontologies, KGs, and semantic data schemata to provide practicable technological solutions for going FAIR, we introduce and discuss the CLEAR Principle in reference to KGs. However, the main principles underlying CLEAR are independent of semantic technologies and can be applied to other information management systems.

In the *Utilizing semantic units as FAIR Digital Objects constitutes a strategy for attaining CLEAR* section, we provide a brief introduction to the concept of **semantic units**, and in the section *How semantic units make knowledge graphs CLEAR*, we discuss how KGs organized into semantic units and corresponding FDOs meet the criteria on data and metadata specified in the CLEAR Guiding Principle, resulting in **FAIR and CLEAR KGs**. We also discuss how the organization into semantic units facilitates the development of new user interfaces that support different exploration strategies that increase the human explorability and cognitive interoperability of data and metadata.

In the *Conclusion* section, we discuss the contributions that semantic units and the CLEAR Guiding Principle potentially provide for solving the three challenges introduced in the *Problem statement* section and how semantic units as FDOs contribute to the bigger picture of going FAIR.

## Problem statement

### Challenge 1: machines need more information than humans for data to be actionable, and humans do not want to look at large and complex graphs

Humans possess an innate ability to deal with contextuality and vagueness of statements using metaphors, metonymies^2^, or general figures of thought that are based on conventions and personal experience. As a result, humans often have no problem with missing information because they still understand the intended content. Machines, on the other hand, demand explicit information to process data. This contrast becomes evident in scenarios where concise statements, imbued with contextual nuances, suffice for human communication, but machines require comprehensive and explicit data representations. For instance, someone living in New York has no difficulties understanding the following dialogue [25]:

A: How did you get to JFK airport?
B: I stopped a cab.

Taken literally, however, the dialogue makes no sense because *B* would not have left the spot just by stopping a taxi. An untrained machine would have substantial difficulties identifying the implied meaning, because important contextual information is missing. A human reader, on the other hand, understands what has been said as referring to a well-known general figure of thought.

One consequence of being experts in dealing with contextuality, vagueness, and missing information is that most humans prefer statements to be concise, making communication more time-efficient, implying additional information via context, and by the sender of the information relying on the receiver to possess the relevant background knowledge to correctly decipher the information. In the end, efficient communication between people is only possible through this metonymic understanding of language [25].

**Fig. 1:**
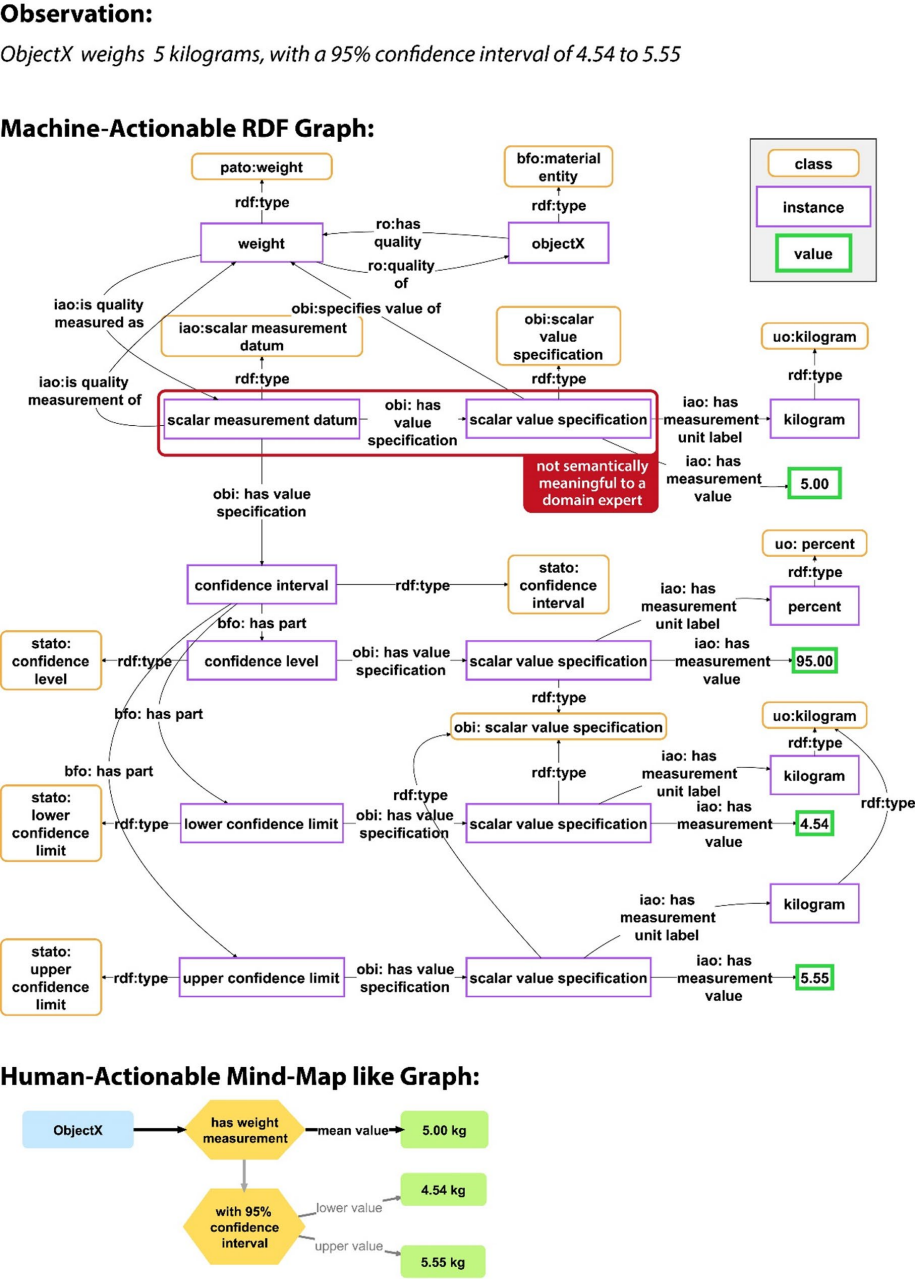
Comparison of a human-readable statement with its machine-actionable representation as a semantic graph following the RDF syntax, and with its human-actionable representation as a mind-map like graph. Top: A human-readable statement about the observation that objectX weighs 5 kg, with a 95% confidence interval of 4.54 to 5.55 kg. Middle: A representation of the same statement as a graph, using RDF and following the general pattern for measurement data from the Ontology for Biomedical Investigations (OBI) [26] of the Open Biological and Biomedical Ontology (OBO) Foundry. Marked red is an example of a triple statement that is not semantically meaningful for a domain expert. Bottom: A representation of the same statement as a mind-map like graph, reducing the complexity of the RDF graph to the information that is actually relevant to a human reader

Besides the need to make all required information explicit for machines, choices for modelling data are frequently influenced by considerations regarding how to best query specific information and how to make semantic data schemata more reusable, which may result in an increase in the complexity of the graph to modularize and standardize graph patterns. Therefore, when human-readable statements are being translated into machine-actionable and easy to query representations that follow, for example, the RDF triple syntax paradigm of *Subject-Predicate-Object*, these graph-based representations are often much more complex than human readers like them to be, often containing triples that are not semantically meaningful for a domain expert (e.g., Fig. 1).

We are thus dealing with a dilemma that arises from the conflict between machine-actionability and human-actionability: **the more we push data representations towards machine-actionability, the more complex and thus the less human-actionable they become.** Humans do not want to see large and complex graphs, because these graphs usually contain lots of information that is irrelevant for humans and that, in bulk, reduces the graph’s comprehensibility. This dilemma represents an impedance mismatch.

If we want to make the data and metadata of KGs FAIR for both machines *and* humans, we need to make them easier for humans to comprehend. In other words, we must increase the cognitive interoperability of data and metadata, for instance by providing new means of exploring and navigating the graph in the form of a mind-map like graph (e.g., Fig. 1, bottom), filtering out information that the user is currently not interested in or that is only required for their machine-actionability, and by providing multiple entry-points for exploring the graph, zooming in and out across different levels of representational granularity.

### Challenge 2: graph query languages are entry barriers for interacting with knowledge graphs

The typical KG is either based on RDF/OWL and stored in a tuple store, or it is a labeled property graph stored using, for instance, Neo4j. Directly interacting with the graph to add, search for, update, or delete data and metadata requires the use of a graph query language. For RDF/OWL, this is, for example, SPARQL, and for Neo4j it is Cypher.

Graph query languages allow detailed and very complex queries but **writing queries in SPARQL or Cypher is demanding**. Someone without experience in writing such queries will have a hard time finding the data and metadata they are interested in. And many software developers are also not familiar with graph query languages and struggle with their complexity when attempting to learn them. Thus, having to write queries with a graph query language represents an **entry barrier** and therefore substantially limits the human-actionability of data and metadata stored in KGs [27].

There is a growing research interest in using large language models (LLMs) and other AI-based systems for mitigating these problems, as they have the capacity to transform natural language questions into SPARQL queries, thereby enhancing the accessibility of KGs by eliminating the necessity to learn the complex query syntax and increasing the efficiency of searches by reducing the time and effort required for constructing queries [28]. However, the use of LLMs in constructing queries can lead to misinterpretation of natural language questions due to the ambiguity present in natural language expressions, resulting in inaccurate or unintended outcomes [29]. Additionally, LLMs have been observed to demonstrate proficiency in handling straightforward queries but encounter challenges with intricate or nested queries [30], necessitating manual refinement, and their accuracy is also contingent on the quality and scope of their training data, which might limit their performance in scenarios involving domain-specific terminology or uncommon query structures.

Technical solutions to mitigate these issues are required, such as openly available and reusable query patterns that link to specific graph patterns. It also would be helpful if users could specify queries in a way that they look like mind-maps without having to use the formalization of a graph query language. The underlying semantic data schemata for such query graphs and their translation into SPARQL or Cypher queries, however, would have to be provided by the KG application and not by the users.

### Challenge 3: knowledge graphs often do not support contextual exploration and visual information seeking strategies

In their search for specific data and metadata, users of KGs are often confronted with the task to find the needle in the haystack, especially when dealing with large and complex KGs. Finding specific information within the vast sea of data can be challenging, and effective data structures and easy to use user interfaces are essential to support the users’ needs. To this end, two main data exploration tasks must be distinguished based on different user motivations for accessing data and metadata in a KG [31]:

1. **Known-item search**: Users have a well-defined information need and a clear idea of the expected results (*conventional search* [32]), and aim to find a specific set of resources or relations in the KG that satisfy this need (where they run into *Challenge 2*).
2. **Browse**: Users seek to develop a general understanding of the KG’s contents or discover unexpected patterns within it. Such **exploratory search** requires an exploration effort and is usually open-ended, has unclear information needs, and is often used for learning and exploration tasks [33].

In practice, users often combine these two tasks, because they desire to continue their exploration of the KG from their initial access point in semantically meaningful ways, using general overviews and relevant contextual information to facilitate efficient navigation.

**Fig. 2:**
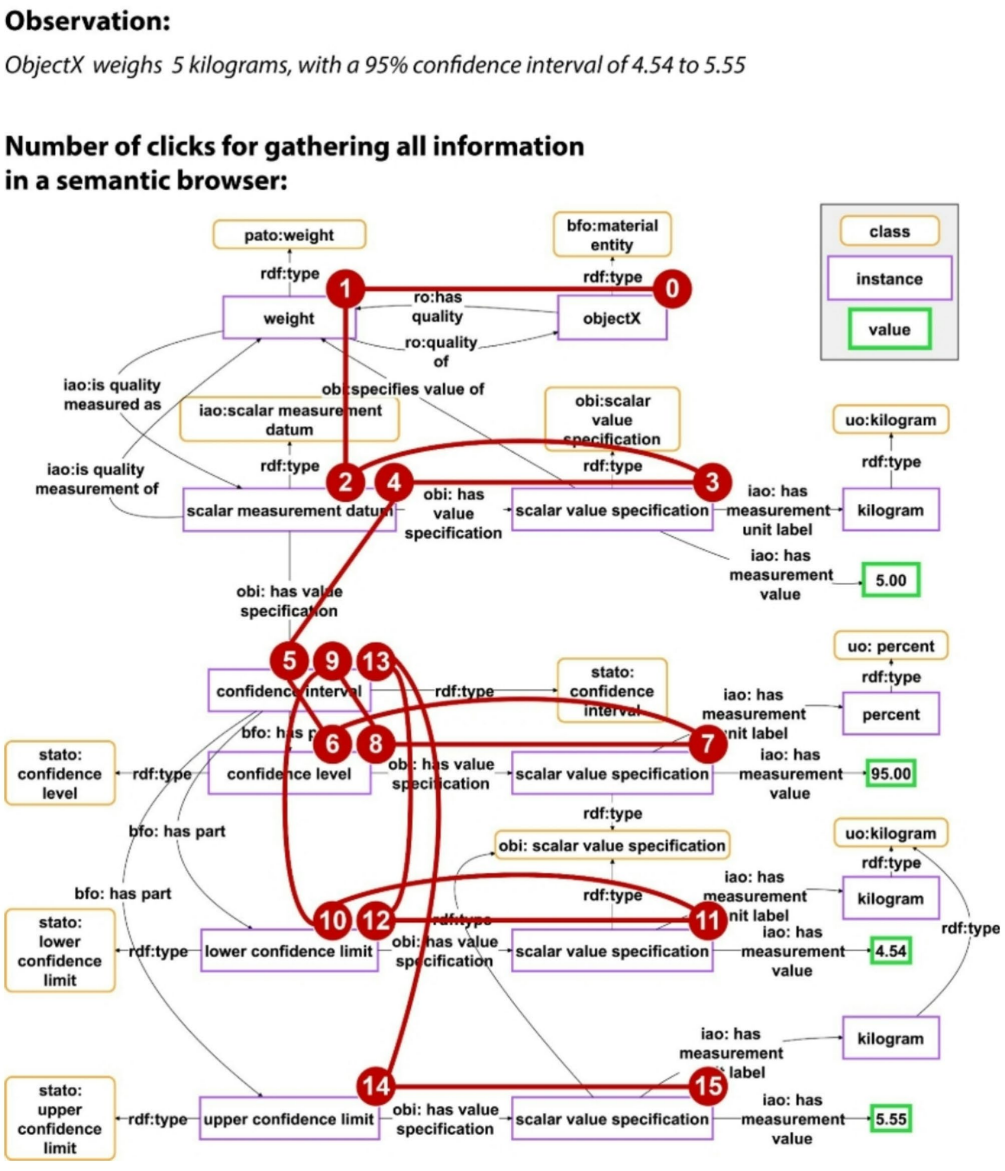
Steps required for exploring the representation of an observation in a knowledge graph using a semantic data browser. Top: A human-readable statement about the observation that objectX weighs 5 kg, with a 95% confidence interval of 4.54 to 5.55 kg. Bottom: A representation of the same statement as a graph (see also Fig. 1). The numbers in the red circles and the red lines indicate the number of clicking-steps and the path that a user must follow to collect all the information about the observation in the knowledge graph, when using a semantic data browser

A **semantic data browser** allows users to start exploring the graph from a single resource as the entry point, and moving from here along triple paths following RDF links [33, 34]. For the graph shown in Fig. 1, with a semantic data browser, a user would have to click 15 times to gather all the information associated with the observation (Fig. 2). Although a semantic data browser facilitates path-based exploration following RDF links, it frequently poses challenges and confuses users because of the high complexity of machine-actionable data representations, with many added resources that are not relevant for a human reader to understand the meaning (see *Challenge 1*; Fig. 1 Middle). The alternative of providing KG visualizations that display the content of a KG as a graph with nodes and edges, however, is not preferable. Graph visualizations often include irrelevant parts of the graph and are not scalable with an expanding KG, resulting in excessive complexity and information density, even for small graphs [35, 36]. The larger the KG becomes, the likelier it is that even domain experts find its contents to be increasingly hard to grasp, requiring exploratory tools to support them in comprehending the contents.

Regarding exploratory search, information technology systems must facilitate complex tasks that users wish to accomplish, including fact-finding, understanding cause-effect chains, or understanding controversial topics [37]. Respective data exploration strategies should ideally promote not only knowledge utility (i.e., increase a user’s domain knowledge) but also exploration experience (i.e., provide a user with a positive and pleasant exploration experience) and ultimately enhance exploration effectiveness [33]. These requirements align with **visual information seeking mantras** such as:

1. **Overview first, zoom and filter, then details-on-demand** [38]:
  1.1. Gain an overview of the contents of the entire information technology system,
  1.2. zoom in on data points and relationships of interest and filter out everything that is not of interest, and
  1.3. select a data point, a relationship, or groups of them and get details about them when needed.
2. **Search first, show context, and expand on demand** [39]:
  2.1. Search for a specific data point,
  2.2. show relevant contexts given the user’s current interests
  2.3. so that users can expand contexts in the directions they are interested in.
3. **Details first, show context, and overview last** [40] **or overview for navigation**:
  3.1. Start with a specific data point,
  3.2. show relevant contexts given the user’s current interests
  3.3. so that users can explore these contexts in detail, using an overview for navigation purposes.

The *‘Overview first’* strategy provides an intuitive guideline to the interaction requirements between the user’s need for a broad awareness of the entire information space and all of its items (*seeing the entire collection* [38]) and the needs for seeing details, which is useful when dealing with datasets of moderate size [41]. Conversely, the *‘Search first’* strategy is optimized for large datasets, where providing a comprehensive overview for top-down analyses is often not feasible. It accounts for the needs of researchers who are not interested in general knowledge overviews or global patterns in the data, but seek answers to specific questions about one or several specific data points [41] and relates to known-item search. The strategy is similar to online map search strategies, where search results provide the starting points for exploring local neighborhoods [42] and where *‘context’* is understood as some sort of localized overview. The *‘Details first’* strategy is designed for data such as spatial data, where experienced users know precisely where interesting data points lie and need support in exploring from there [40], with overviews serving as navigation aids.

In optimizing the explorability of information technology systems, insights from all three mantras can be leveraged to enable zooming in and out of specific contexts and data points, facilitating information filtering, and providing overviews for effective navigation. These strategies contribute to enhancing the contextual explorability capabilities of human users within the systems, promoting cognitive interoperability, and ultimately advancing data-driven research and analysis across domains.

Unfortunately, many KGs do not support any of these exploration strategies. In the past, the attention in KG research was more on KG construction than on the investigation and visualization of the graph itself, and consequently not many tools supporting KG exploration have been developed [33] (for early approaches facilitating exploratory searches focusing on textual or visual interfaces, see [43, 44]; for new approaches based on path parameters, exploration paths, and knowledge anchors, see [33]).

It has been shown that successful implementations of exploratory search strategies expand a user’s domain knowledge when exploring unfamiliar domains (i.e. knowledge utility) [34, 45], and approaches supporting the three exploration strategies exist for KGs:

**KG profiling and summarization** supports users in gaining a **high-level overview of the graph** and therefore assists users in initial exploratory stages [46]. In the context of KGs, this could be the number of classes and their instances and value distributions for specific properties [47], or word clouds of resource labels, supporting users in gaining a general overview [48]. Structural summarization [49, 50] and pattern mining [51, 52] provide either a compact representation of the main features of the graph or create a somewhat simplified graph derived from the original graph, offering context to a given data point or an overview of the typical relationships of entities in the KG.

**KG exploratory search** supports users in **identifying and exploring entities and relations of interest in the graph**, providing contextual knowledge that informs next exploration steps. It refers to the open-ended exploration effort that combines browsing and searching for knowledge acquisition [53], often motivated by a vague information need [46]. It can be supported by approaches such as search-by-example [54, 55] or domain-specific query interfaces that support users in specifying SPARQL queries without having to write SPARQL [56, 57]. Faceted exploration allows users to select facets as filters to decrease the size of the graph to what is relevant [58–60]. KG search interfaces that are based on facets can also be developed in such a way that they can be used for gradually formulating complex analytic queries without having to write SPARQL [61].

**KG exploratory analytics** provide **specific views on the graph**, aggregating data into meaningful and relevant data collections tailored to serving specific data needs. This is an iterative process of data discovery and analytical querying of data which are not well known to the users [46], requiring multidimensional analysis functionalities over KGs that are typical of relational data warehouses, by describing multidimensional and statistical information within the KG [62, 63].

Although all these concepts facilitate contextual exploration of KGs, they are not sufficient and typically represent solutions that are tailored to a specific use case and thus cannot be directly transferred to other domains and use cases. We need innovative general exploratory methods and tools that enhance human explorability of data in a KG.

## The CLEAR Principle

The effective utilization of KGs to enhance the machine-actionability and interoperability of data and metadata has garnered significant attention across various domains. However, the inherent challenge lies in striking a delicate balance between accommodating the needs of machines and catering to the needs and the cognitive capabilities of human users of KGs and thus domain experts, data scientists, and software developers. We contend that to effectively address this challenge, a paradigm shift is imperative, necessitating an extension to the EOSC Interoperability Framework and comprehending the FAIR Guiding Principles with the CLEAR Principle.

### Cognitive interoperability

Semantic interoperability plays a central role in enabling the effective exchange of data and metadata. This encompasses both human-machine and machine-machine interactions, aiming to ensure seamless and actionable communication. According to EOSC, semantic interoperability is achieved *“when the information transferred has*,* in its communicated form*,* all of the meaning required for the receiving system to interpret it correctly”* [6] (p. 11). However, it warrants consideration whether the four layers of the EOSC Interoperability Framework [6]―technical, semantic, organizational, and legal interoperability―and the four principles of FAIR, with their 15 associated criteria, are sufficient in ensuring the effective communication of data and metadata between humans and machines and the human-actionability of data and metadata.

Regarding human-actionability, it is important to know which actions hold significance for users when interacting with data and metadata. While users want to reliably and exhaustively find all data they are interested in within a KG and frequently also want to integrate data from different KGs, all of which requires the data’s semantic interoperability, above all, users primarily seek to correctly comprehend the data—human-interpretability of data is a prerequisite for their human-actionability. Comprehending data goes beyond being able to correctly understand the meaning of individual data points and includes also being able to contextualize them and explore further data points from any given data point in semantically meaningful ways. In essence, it involves understanding the interconnections between different data points. This intricate level of comprehension mandates data to possess cognitive interoperability, enabling users to extract meaningful insights effectively.

**Cognitive interoperability** is a critical characteristic of data structures and information technology systems that plays an essential role in facilitating efficient communication of data and metadata with human users. By providing intuitive tools and functions, systems that support cognitive interoperability enable users to gain an overview of data, locate data they are interested in, and explore related data points in semantically meaningful and intuitive ways.

The concept of cognitive interoperability encompasses not only how humans prefer to interact with technology, i.e., **human-computer interaction**, but also how they interact with information, i.e., **human information interaction**, considering their general cognitive conditions. In the context of information technology systems such as KGs, achieving cognitive interoperability necessitates tools that increase the user’s awareness of the system’s contents, aid in understanding their meaning, support data and metadata communication, enhance content trustworthiness, facilitate integration into other workflows and software tools, and clarify available actions and data operations.

Additionally, cognitive interoperability also encompasses ease of implementation of data structures and their management for developers and operators of information technology systems. It thus addresses the specific data, tool, and service needs of the three main personas [64] identified for users of information management systems such as KGs, namely **information management system builders** (i.e., information architects, database admins), **data analysts** (i.e., researchers, data scientists, machine learning experts), and **data consumers** (i.e., stakeholders, end users, domain experts).

Knowledge representation should always accommodate cognitive limitations of human users, and data and metadata standards should support their cognitive interoperability. Cognitive interoperability should be considered when developing user interfaces, for instance by including language-specific labels for multiple languages, including synonyms in data and metadata representations [65] and utilizing them in searches, considering the fact that humans can hold only 5–9 items in memory [66], and that search tasks can differ in complexity (e.g., fact-finding, understanding cause-effect chains, or understanding controversial topics) [37]. As information technology systems grow in complexity and size, innovative exploratory methods and tools [46, 67] become crucial to enhance **human explorability of data** and are important to improve the general interoperability between data and the cognitive capabilities of human users. This can be accomplished by **reducing the complexity of the information displayed** in the system’s user interface to what currently interests a user, and by providing users semantically meaningful ways to **contextually** access and explore the graph from any given data point. We thus argue that cognitive interoperability represents an essential aspect of the general interoperability of data and metadata, and we therefore suggest adding cognitive interoperability as a fifth layer to the four layers identified by the EOSC Interoperability Framework.

### Complementing FAIR with the CLEAR Principle

Because human thoughts are combinatorial (simple parts combine) and recursive (parts can be embedded within parts), breathtaking expanses of knowledge can be explored with a finite inventory of mental tools. ([68], p.360)

To address the challenges of achieving cognitive interoperability and overcoming the three challenges identified above (see *Problem statement*), the **CLEAR Principle** for **C**ognitively interoperable, semantically **L**inked, contextually **E**xplorable, intuitively **A**ccessible, and human-**R**eadable and -interpretable data and metadata is introduced (see Box 2). The CLEAR Principle complements the FAIR Guiding Principles, with the goal of striking a balance between accommodating the needs of machines and the needs of human users, and thus between the needs for machine-actionability and human-actionability of data and metadata. Whereas the FAIR Guiding Principles place a strong emphasis on the machine-actionability of data and metadata, the CLEAR Principle with its emphasis on cognitive interoperability and human explorability do that for the human-actionability of data and metadata, drawing inspiration from fundamental principles of human cognition. Improving human explorability and overall cognitive interoperability of data and metadata in FAIR information technology systems turns them into **FAIR and CLEAR information technology systems**.

In order to support the abovementioned exploration strategies, data and metadata must be organized into semantically meaningful but interlinked subsets, each uniquely represented by a GUPRI, enabling easy referencing and identification of each subset. Ideally, **each subset is organized as an FDO** and is classified as an instance of a semantically defined **FDO class**, documented in a corresponding ontology or controlled vocabulary (Box 2: C1). Subsets can be **(recursively) combined** to encapsulate complexity into manageable units, forming subsets of coarser granularity (Box 2: C2) which, ideally, are also organized as FDOs instantiating corresponding FDO classes. The organization of a dataset into such subsets results in a significant increase in the human-explorability of its data and metadata. Supporting human explorability of data also aims at enhancing the flexibility of knowledge management within information technology systems and overall enhancing the expressivity of such systems.

Within KGs, this can be achieved by structuring the graph into separate, semantically meaningful subgraphs, which can be recursively combined to form larger subgraphs, with each subgraph being represented in the KG by its own resource and organized as an FDO.

In human communication, **propositions** are the smallest units of semantically meaningful information, conveyed through statements relating proper names and kind terms^3^. The RDF framework embodies statements as triples, with individual entities, concepts, and their connections represented as instance, class, and predicate resources, respectively. However, natural language statements often comprise more than one object and thus involve **n-ary relations**, which cannot be captured within a single triple, such as the statement *“Peter travels by train from Berlin to Paris on May 25th 2024”*. Therefore, RDF triples frequently map to natural language statements in a many-to-one instead of a one-to-one relation. Consequently, in a KG, simple statements featuring a single verb or predicate are modeled using one or more triples and constitute the smallest type of semantically meaningful subgraphs (cf. Figure 1).

Understanding **human-actionable statements** rather than single RDF triples as the **main units of communication** and thus as **first-class citizens** in a KG is essential when optimizing user interfaces for cognitive interoperability. Every datum in a dataset can be viewed as an individual statement, either explicitly or implicitly through its contextualization (e.g., as a list of measurement values in a column of a table that is interpreted as weight measurements with *kg* as their unit). By organizing triples that model a particular statement in their own subgraph, a KG can be organized into a set of subgraphs each of which maps to a particular statement (Box 2: C1). Such statement subgraphs can be organized into **nanopublications**^4^ [69–71] and would form statement FDOs.

A **statement FDO** represents a fundamental unit of information, comprising the smallest, most granular unit of information. It takes the form of a particular proposition, such as a volume measurement. Tabular data structures can be organized similarly by for instance structuring statements as single rows in a table, with each row having its own GUPRI that refers to the entire statement and that defines a corresponding statement FDO.

In addition to having its own GUPRI and the typical FDO metadata, each statement FDO should:

1. provide a specification of who created the FDO and distinguish it from who authored its content;
2. reference the schema GUPRI for the data schema (i.e., SHACL shape, table structure, etc.) that was used for modelling the statement, to support schema interoperability [65];
3. specify the formal logical framework, if any, that was used for modelling the statement to indicate whether the FDO’s content supports reasoning and which logical framework must be used to support logical interoperability [65];
4. specify the statement category, distinguishing assertional statements (e.g., *Swan Anton is white*) from contingent (e.g., *Swans can be white*), prototypical (e.g., *Swans are typically white*), and universal statements (e.g., *Every swan is white*); and
5. provide a human-readable representation of the statement to increase its cognitive interoperability.

Fine-grained statement FDOs can then be combined in an information technology system to form coarser-grained **nested FDOs** (Box 2: C2) that organize statements into semantically meaningful collections of statements, forming respective coarser-grained FDO types, which can be organized into *Research Object Crates*^5^ (RO-Crates) or similar technical implementations. Each such nested FDO should instantiate a corresponding FDO class that specifies the type of FDO, and different nested FDO types should be distinguished.

Organizing data and metadata into FDOs of various types leads to a nested structure of data and metadata, representing **different levels of representational granularity**. Additional types of subsets/subgraphs and corresponding FDO types can be identified based on **contextual differences** and inherent **hierarchies**, such as taxonomies and partonomies. Organizing a graph or a dataset into semantically meaningful subgraphs or subsets and structuring them into corresponding FDOs with accompanying metadata not only contributes to their cognitive interoperability and general reusability but also their propositional interoperability [65].

Besides these structural aspects of organizing data and metadata into different types of semantically meaningful FDOs, explorability also requires adequate tools and easily understandable interfaces that provide operations that support users in pursuing different exploration strategies. We need tools and interfaces that **decouple the human-readable display of data and metadata from their machine-actionable storage** (Box 2: C3). The organization of data and metadata into different types of FDOs should thereby facilitate the development of user interfaces that support new ways to explore data and metadata, including **mind-map-like graphical** user interfaces in addition to **form-based textual** user interfaces. By reducing the complexity of data and metadata displayed in the user interface to what is relevant for human comprehension, while disregarding information that is only relevant to machines (for an example, see highlighted triple in Fig. 1), mind-map-like displays enhance human-actionability of data and metadata. They can also provide graphical visual representations of complex interrelationships between various entities that are easier to comprehend than from their textual representations—just think of trying to understand a textual representation of a family tree as opposed to its graphical representation. Integrating textual and graphical displays within the user interface further promotes user-friendly interactions with the data.

Moreover, information technology systems and their user interfaces should utilize the modular structure of the graph or dataset being structured into semantically meaningful FDOs and allow users to zoom in and out of data displays and **provide contextual information** for data points currently in focus for data exploration. The user interfaces should also support **making statements about statements** intuitively, as well as allowing users **to specify query graphs** using form-based or mind-map-like interfaces **without requiring knowledge of query languages**.

All these considerations result in the formulation of the CLEAR Principle, which offers significant potential to enhance cognitive interoperability of data and metadata and addresses existing challenges of information technology systems (for the specification of the CLEAR Principle, see Box 2).

**Box 2:** The CLEAR Principle with its three criteria

C1	(meta)data are structured into semantically meaningful statement subsets, each modelling an individual proposition that is represented by its own globally unique and persistent identifier (ideally forming a statement in the form of a statement FAIR Digital Object) that enables its independent actionability, its referencing, and its identification, and that instantiates a corresponding semantically defined (statement FAIR Digital Object) class
C2	(meta)data statements and other compound subsets can be (recursively) combined to form a compound subset (i.e., a nested FAIR Digital Object), each with its own globally unique and persistent identifier, instantiating a corresponding semantically defined (nested FAIR Digital Object) class
C3	human-readable textual and graphical display of (meta)data is decoupled from machine-actionable (meta)data storage to reduce the complexity of (meta)data displayed to human readers and to display only information that is relevant to human readers, and a user interface supports contextual exploration of the (meta)data

## Utilizing semantic units as FAIR digital objects constitutes a strategy for attaining CLEAR

**Fig. 3:**
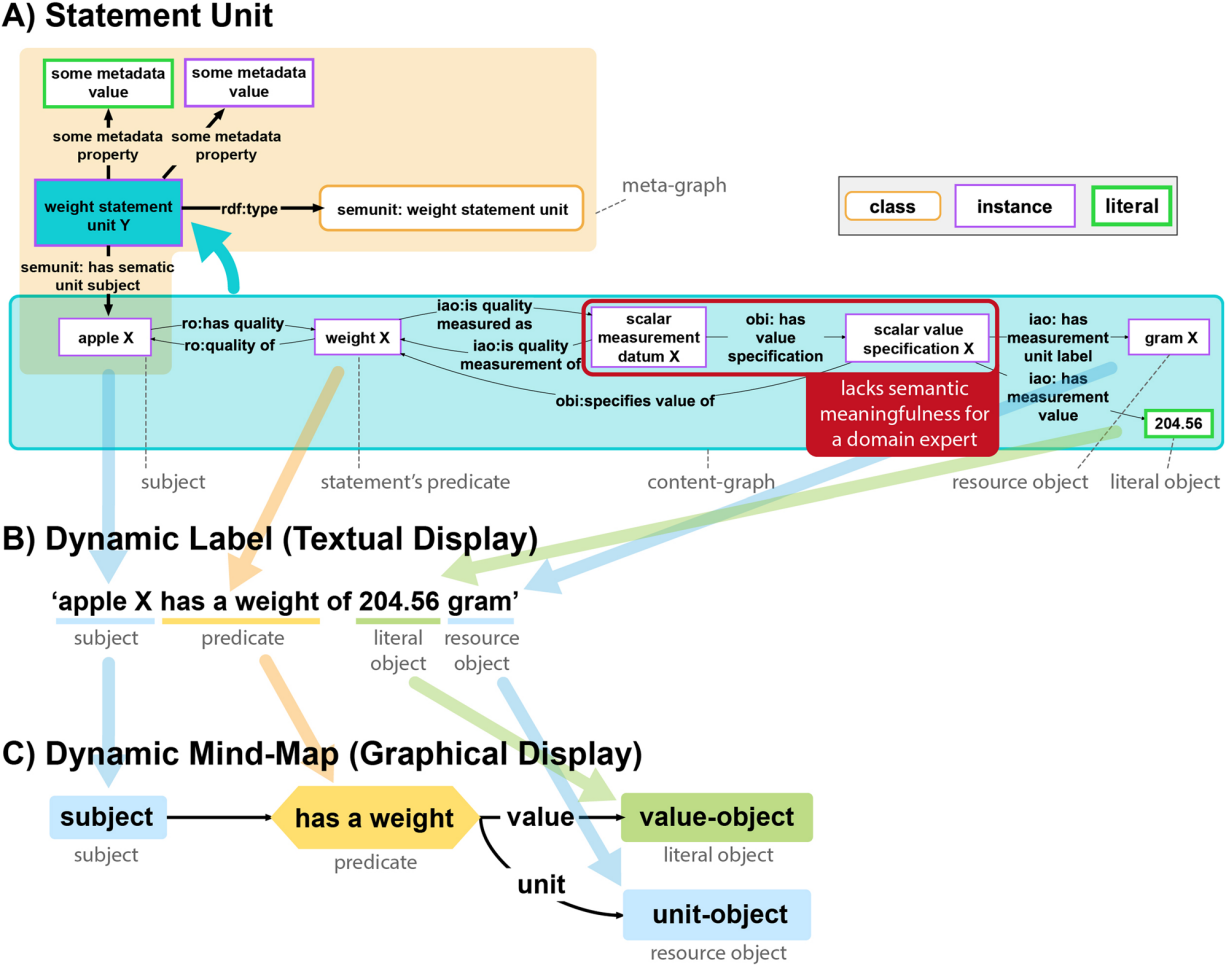
Example of a semantic unit. **A**) The statement *“Apple X has a weight of 204.56 grams”* modelled in RDF/OWL, organized as a statement unit, which is a specific type of semantic unit. The content-graph, denoted within the large blue box, articulates the statement with ‘apple X’ as its subject and ‘gram X’ alongside the numerical value 204.56 as its objects. The peach-colored box encompasses its meta-graph. It explicitly denotes the resource embodying the statement unit (bordered bright blue box) as an instance of the *SEMUNIT: weight statement unit* class. Notably, the Globally Unique Persistent and Resolvable Identifier (GUPRI) of the statement unit (*‘SEMUNIT: weight statement unit’*) is also the GUPRI of the semantic unit’s content-graph (the subgraph in the large blue box). The meta-graph also contains various metadata triples, here only indicated by *some metadata property* and *some metadata value* as their placeholders. Highlighted in red within the content-graph is an example of a triple that is required for modelling purposes but lacks semantic meaningfulness for most domain experts. The dynamic label **B**) and the dynamic mind-map **C**) associated with the statement unit class (*SEMUNIT: weight statement unit*). [Figure modified from [73]

Enhancing the cognitive interoperability of a KG to elevate it to a CLEAR KG and to address the three challenges discussed in the *Problem statement* requires a specific organization of the KG and its data (see Box 2). Our previous work on **semantic units** [72, 73] provides a framework that offers the required organization by structuring a KG into identifiable sets of triples and thus **subgraphs**. Unlike conventional methods of partitioning a KG [74–82], the semantic units approach focuses on structuring the graph into units of representation that are semantically meaningful and easily comprehensible to human users of the KG (Fig. 3). In the following, we provide a summary of that semantic units framework (for more details on semantic units, please refer to [72, 73]).

Technically, a semantic unit is a subgraph within the KG. It is represented in the KG with its own resource, designated as GUPRI that identifies the associated subgraph. Consequently, referring to a semantic unit resource via its GUPRI is equivalent to referring to the contents of its content-graph, empowering KG users to make statements about the content encapsulated within the semantic unit’s content-graph.

Organizing a KG into semantic units introduced a new layer of triples into the graph. We call the existing graph the content-graph layer, and all newly added triples constitute the meta-graph layer of the KG. Each semantic unit resource, together with all triples that have a semantic unit resource in their *Subject* or *Object* position, belongs to the meta-graph layer. Analogously, we can distinguish a content-graph and a meta-graph part for each semantic unit (Fig. 3A). Consequently, merging the content-graphs of all semantic units of a KG results in the content-graph layer of that KG.

Every semantic unit resource instantiates a corresponding **semantic unit class** [Criteria 1,2], which includes a human-readable description of the type of information covered by its instances and by their associated content-graphs. Semantic unit resources introduce a new type of representational entity, augmenting the expressivity of KGs beyond instances, classes, and relations (i.e., RDF predicates). Semantic units can be accessed, searched, and reused as identifiable and reusable data items, forming units of representation that allow their implementation as FDOs in the form of nanopublications [72] or RO-Crates using RDF/OWL-based graphs (or any other technical implementation such as labelled property graphs or tabular data structures [73].

### Statement units

Different categories of semantic units can be distinguished [72, 73]. A statement unit represents the **smallest**,** independent proposition that is semantically meaningful for a human reader** [Criterion 1]. Depending on the arity of the underlying statement, the content-graph of a statement unit comprises one or more triples (Fig. 3A, large blue box). Technically, this content-graph is organized in its own Named Graph, the GUPRI of which is also the statement unit resource. Alternatively, in a labeled property graph such as Neo4j, the GUPRI of the statement unit resource would be assigned to all nodes and relations belonging to this unit via a respective property-value pair. Structuring a KG into statement units mathematically partitions the KG, ensuring that each of its triples belongs to precisely one statement unit.

By assigning to each statement unit class a specific graph schema that specifies how statement units of that type must be modeled, for instance in the form of a SHACL shape specification [83] that possesses its own GUPRI and includes constraints for its slots, semantic units also support schema interoperability [65]. By including a specification of the logical framework that has been applied (e.g., Description Logics or First-Order Logic), logical interoperability [65] can be specified for all statement units that are based on the same logical framework.

**Dynamic labels** provide human-readable textual representations and **dynamic mind-maps** graphical representations of statements, facilitating the display of complex n-ary propositions in a concise and understandable manner, ignoring information that is only relevant for the machine but not meaningful to a user (Fig. 3B, C). Each statement unit class has its associated SHACL shape and templates for dynamic labels and mind-map patterns. The use of dynamic labels and dynamic mind-map patterns results in a clear separation of machine-actionable storage from human-actionable display of data and metadata in a CLEAR KG [Criterion 3], which significantly enhances the content’s cognitive interoperability. It allows exploring the contents of a CLEAR KG at the level of human-readable statements, where a statement that may be modeled in RDF with more than 20 triples could be represented in a single statement unit with a human-readable dynamic label or mind-map, therewith reducing the complexity of the graph by filtering out information that is only relevant to machines and of no interest to a human reader (see *Challenge 1*). This also supports the development of user interfaces that understand statements as the main units of communication, treating **statements as first-class citizens** of the KG.

Statement units can be organized into **nanopublications** [72] (or any other technical implementation) and could provide a framework for **statement FDOs**. Several subcategories of statement units can be distinguished, including a user-friendly formalism for negations, and they can be organized in a taxonomy of statement unit classes (for a detailed discussion of various types of statement units, see [73]), which can contribute to a general classification of corresponding statement FDOs. Structuring a KG into semantic units thus substantially increases its overall expressivity.

### Compound units

**Fig. 4:**
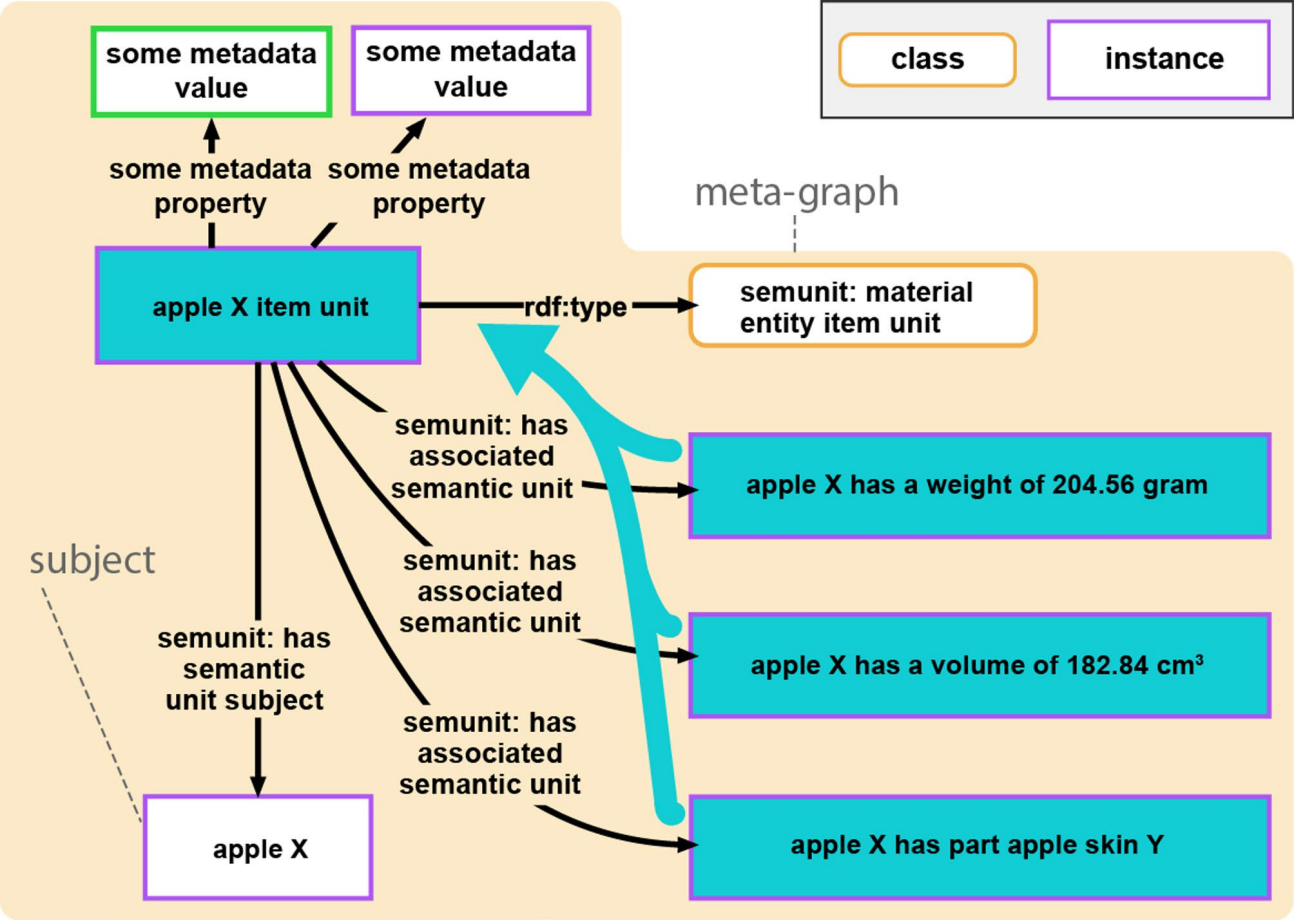
Example of a compound unit that comprises several statement units. Compound units possess only indirectly a content-graph, through merging the content-graphs of their associated statement units. The compound unit resource (here, *‘apple X item unit’*), however, stands for these merged content-graphs (indicated by the blue arrow). Compound units possess a meta-graph (shown in the peach-colored box), which documents the semantic units that are associated with it. [Figure taken from [73]

Compound units facilitate the organization of statement units and other compound units into larger, semantically meaningful subgraphs (Fig. 4). A compound unit can be viewed as a container of a collection of associated semantic units. Merging the content-graphs of its associated semantic units constitutes the content-graph of the compound unit. Analog to statement units, each compound unit is a semantic unit that is represented in the graph by its own resource and thus possesses its own GUPRI, and it instantiates a corresponding compound unit ontology class [Criterion 2]. Compound units can be organized into RO-Crates (or any other technical implementation) and could provide a framework for **nested FDOs** and thus types of FDOs that are coarser-grained than statement FDOs. Several subcategories of compound units can be distinguished, each serving a specific purpose in knowledge organization [72, 73], resulting in a classification of compound units that can contribute to the general classification of nested FDOs.

Different types of compound units can be distinguished (for a detailed discussion of various types of statement units, see [72, 73]). An **item unit** [72], for instance, is a compound unit that associates all statement units of a CLEAR KG that share the same subject resource, and this subject resource also serves as the subject of the item unit [criterion 2]. They represent semantic units at the next coarser level of **representational granularity**, above the level of statement units. An **item group unit** [72] is a compound unit comprising at least two item units that are semantically linked through statement units sharing the same subject as one item unit and one of its objects as the subject of another item unit. Through such linking statement units, a chain of interconnected item units can be formed that can be represented in an item group unit, forming a semantic unit at the next coarser level of representational granularity, above the level of item units. A **granularity tree unit** [72], on the other hand, is a compound unit consisting of two or more statement units that collectively form a granularity tree [76–86]. Any type of statement unit that is based on a partial order relation such as parthood, class-subclass subsumption, or derives-from, can give rise to a corresponding granularity perspective [87–89], of which a particular granularity tree is an instance. Statement units associated with a granularity tree unit are interlinked following a tree-hierarchy, where one statement unit’s object serves as the subject of another statement of the same statement type.

### Question units

**Fig. 5:**
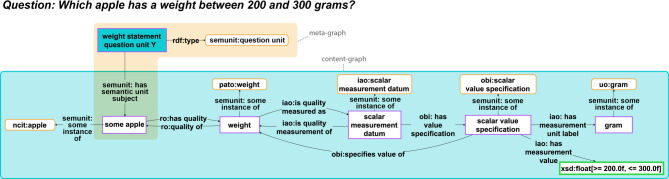
Question units. Example of a question unit. For reasons of clarity, metadata for each semantic unit is not represented

With the introduction of some-instance, most-instances, and every-instance resources, questions can be stored and represented in a KG in the form of question units (see discussion in [73]). A question unit utilizes one or more existing semantic unit classes as their source, creating instances of them as questions that can be documented as a connected subgraph in the KG, akin to other semantic units (Fig. 5).

Based on the SHACL shape specification of the statement unit classes involved, a query-builder can derive corresponding graph queries that can be executed by the KG. If the question unit leaves none of its subject and object position slots underspecified, the question assumes a Boolean *true*/*false* answer. However, by using some-instance and every-instance resources as variables for subject and object resource slots and datatype specifications with underspecified values as variables for literal object slots, respective queries will return a list of matching subgraphs (Fig. 5). For instance, when using a some-instance resource of the type apple (NCIT: C71985) in the subject resource slot in a question unit for weight measurement statement units, the query would return all weight measurement statement units of apples. In the user interface of a KG, the same forms for adding statements units can be used for adding question units.

The implementation of question units that can be represented in the KG and a corresponding query-builder effectively converts searches into objects within the KG, a strategy that bears resemblance to previous works [90, 91] in the field. Overall, the introduction of question units together with corresponding user interfaces and a query-builder would not only provide an intuitively usable framework for specifying SPARQL or Cypher queries without having to know graph query languages (see *Challenge 2*), but could also support ontology development by documenting specific competency questions in the graph [92] in the form of a set of corresponding question units.

### Making statements about statements

The modular and nested organization of semantic units and the fact that each semantic unit in a KG can be represented as an FDO with its own GUPRI opens up new possibilities for making **statements about statements**, introducing an additional layer of communication and metadata enrichment to KGs. Modelling statements about statements in RDF/OWL KGs can be challenging, and several strategies have been suggested, ranging from companion properties, nary relations, Named Graphs, singleton properties, standard reification, and the Blazegraph-specific Reification Done Right, with Named Graphs outperforming the other strategies when conducting more complex queries [93]. Recently, RDF-star [94, 95] has been suggested as an alternative. However, since it only supports making statements about individual triples, it is not suited as a solution for semantic units, where many subgraphs consist of several triples, because the respective statement unit is modelling a non-binary relationship. Consequently, in RDF/OWL KGs, using Named Graphs for organizing semantic units seems to be the best solution.

The representation of semantic units as FDOs with their own GUPRIs facilitates straightforward statement-making about them, with statement units as smallest units of information providing a fine-grained basis and various types of compound units, as collections of statements, more coarse-grained semantically meaningful units of information. Statements about semantic units can themselves be represented in a KG as semantic units and can encompass various aspects, such as associating provenance information with semantic units and indicating compliance with data protection regulations based on, e.g., *GDPR*. Additionally, semantic units being organized as FDOs enable making statements across different databases and KGs, facilitating data sharing and cross-referencing of information [73].

Structuring a KG into semantic units also fosters a communication layer within CLEAR KGs, empowering users to express their own viewpoints and opinions in a structured and machine-actionable manner by adding statements about them to the graph [73].

To realize the full potential of making statements about statements and leveraging its benefits effectively, the development of intuitive user interfaces becomes crucial. By structuring KGs into semantic units, user interfaces can be designed to facilitate making statements about statements. The adherence to a clear and straightforward schema and structure in implementing semantic units further streamlines the development of accompanying user interfaces, tools, and services.

### How to build a CLEAR knowledge graph

In a CLEAR KG, semantic units are implemented as FDOs. These FDOs could take the form of nanopublications for statement units (for a detailed discussion, see [72]) and RO-Crates for collections of semantic units (i.e., compound units). Consequently, RDF/OWL KGs are particularly well-suited for the implementation of semantic units, as nanopublications are based on RDF (for the implementation in Neo4j-based KGs, see [72]).

Following the same strategy for constructing a KG that is organized into semantic units [72], the initial phase of constructing a CLEAR KG entails the identification of statement units as the fundamental information storage FDOs, with compound units serving as nested FDOs. This necessitates the delineation of the statement unit classes indispensable for representing the types of statements essential to the knowledge graph’s scope. Each statement unit class implemented in the KG must be associated with a designated semantic data schema, preferably specified through a SHACL shape specification.

Depending on whether the CLEAR KG is created from scratch, or an existing KG should be transferred, three distinct implementation strategies exist (see discussion in [72]). When built from scratch, a KG application must be developed that organizes incoming information into statement unit FDOs (i.e., nanopublications) in accordance with their designated data schemata. Additionally, the application must have rules established that govern the aggregation of statement unit FDOs into compound unit FDOs, contingent on the specific type of compound unit.

In the event that an existing KG must be transferred to a CLEAR KG, the KG must be restructured into semantic units, with partitioning it into statement units as a first step. This requires the specification of queries based on the semantic data schemata identified in the initial phase that are associated with each statement unit class relevant for the KG’s scope. The queries are used to transfer all triples from the source KG into corresponding statement units for the CLEAR KG.

In circumstances where the restructuring of an entire KG is deemed impractical or undesirable, yet there is a necessity to organize recently integrated information into semantic units, a hybrid approach can be employed. This entails the development of input workflows to ensure that all incoming data aligns with the semantic units’ structure.

Looking at the fact that introducing semantic units requires not only restructuring a KG into multiple Named Graphs but also adding a meta-graph layer that contains additional triples, one may ask how this strategy can enhance the KG’s cognitive interoperability, as the complexity of the resulting CLEAR KG increases. However, while from purely technical triple-based perspective this is certainly true, by treating statements (not triples) as first-class citizens and organizing the entire KG accordingly, the graph becomes more accessible to human users as it is modularized into semantically meaningful units. Moreover, with dynamic labels and mind maps for each statement unit, users do not have to interact with the data structures and thus the triple-layer anymore but can access the information in human-readable and interpretable representations, displayed in a user interface.

## How semantic units make knowledge graphs CLEAR

### Semantic units structure a knowledge graph into different levels of representational granularity, granularity perspectives, and frames of reference

**Fig. 6:**
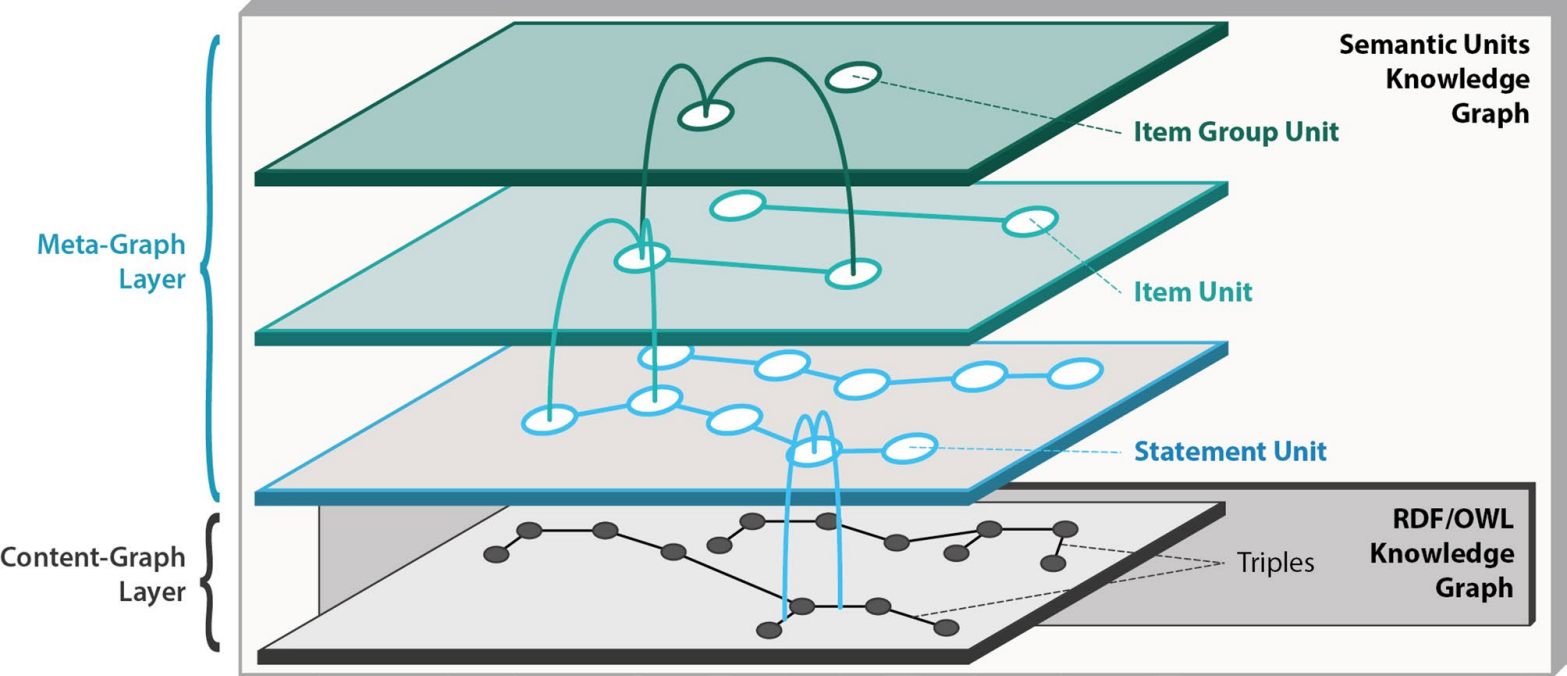
Five levels of representational granularity. The introduction of semantic units to a knowledge graph adds a meta-graph layer to its data graph layer (i.e., content-graph layer), which adds a level of statement units, a level of item units, and a level of item group units to the level of triples and the level of the graph as a whole, resulting in five levels of representational granularity. [Figure adapted from [72]

The semantic units approach provides a powerful organizational framework for enhancing a KG’s overall structure and improving its semantic and cognitive interoperability. By organizing the data graph of a KG into identifiable and semantically meaningful units, a meta-graph layer is created that functions as the **discursive layer** of the KG that comprises **five levels of representational granularity** [72]. At the finest grained level are individual triples, followed by statement units, item units, item group units, and the KG as a whole (Fig. 6) [Criteria 1&2].

The use of semantic units facilitates the visualization of statements in a mind-map-like manner or as natural language statements, greatly aiding human comprehension and exploration of the KG’s contents [Criterion 3]. All statements belonging to an item unit can be displayed on the same user interface page, and the item units belonging to the same item group unit can be represented as a collection of semantically interrelated user interface pages.

The KG’s organization is further enriched by the introduction of granularity tree units present in the graph. Granularity trees organize the content-graph layer and thus the ontological layer of a KG into different **granularity perspectives**, each with its own levels of granularity, providing users with different viewpoints to explore and analyze the KG’s contents effectively. Granularity tree units organize the KG orthogonally to and independent of representational granularity. In other words, while representational granularity organizes the way *how* ontological knowledge is communicated (i.e., the **discursive layer**), granularity perspectives organize the ontological knowledge itself (i.e., the **ontological layer**).

The organization of a KG into semantic units lays the foundation for the development of advanced graphical user interfaces with innovative information-exploration tools, that enable users to access data and metadata in new ways and to rapidly explore them in a user-controlled fashion, with visual representations of data and metadata, thus supporting visual information seeking strategies mentioned above (*‘Overview first’*, *‘Search first’*, and *‘Details first’*). These user interfaces should act as magic lenses, enabling users to find, sort, filter, and present relevant information in user-controlled, intuitive ways.

By leveraging the organization of data and metadata provided by CLEAR KGs, users can swiftly gain an **overview** of the KG’s contents by exploring the graph at the level of item group units, with the possibility to **zoom in** on finer levels of representational granularity, or granularity trees, thereby filtering out data and metadata they are currently not interested in and rapidly getting **details-on-demand**. Granularity tree units with their intrinsic hierarchical organization each structure the data graph of a CLEAR KG into different levels of granularity that, together with the representational granularity of statement, item, and item group units, provide **overviews for navigation purposes** and possibilities for identifying **contexts of interest** to expand upon in directions a user is interested in (see user interface tool example further below).

**Fig. 7:**
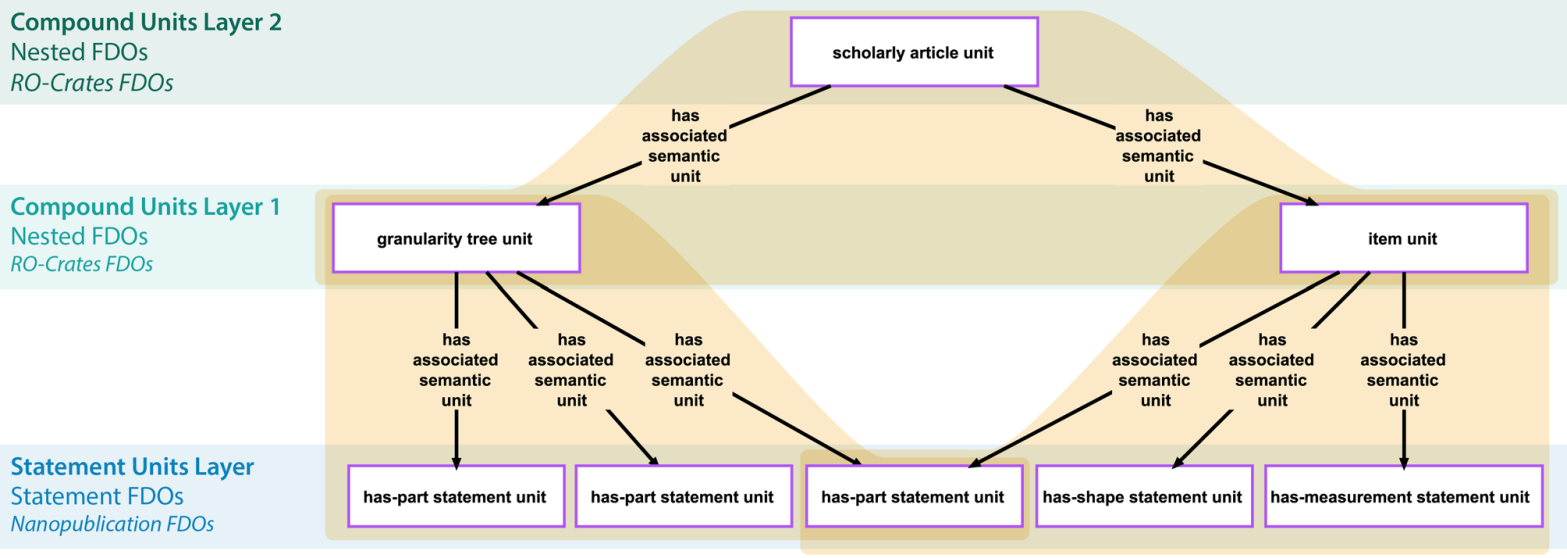
Example of different layers of granularity in a scholarly CLEAR knowledge graph. The knowledge graph contains scientific findings of different scholarly articles. Each article is modelled in the graph using different types of semantic units as FAIR Digital Objects (FDOs). For an article describing the anatomical organization of a particular specimen, the description can be organized into a layer of statement units at the bottom, a layer of compound units consisting of item units and a granularity tree unit in the middle, and a layer of different scholarly article units at the top. More layers can be added depending on the type of information modelled from the scholarly articles

Let us apply this to the representation of the scientific findings of a scholarly article in a CLEAR scholarly KG. As an example, we use a fictional article that describes the anatomical organization of a particular specimen in the form of a hierarchy of has-part relations between anatomical entities, forming a partonomy. Additionally, the description includes shape specifications, length and width measurements, and specifications of positional relationships for some of these anatomical entities (Fig. 7).

The phenotype description can be modelled in the KG as a set of statement units of the types *has-part*, *has-shape*, *has-length*, *has-width*, and *has-positional-relationship-to*, together forming a connected subgraph within the KG. Each of the statement units is organized as a nanopublication and represents a statement FDO. This allows researchers to individually refer to the smallest semantically meaningful units of information of the article, and to make statements about them within the KG.

All statement units that have the same anatomical entity resource in their subject position form an item unit. Each such item unit documents all information from the article that describes this particular anatomical part of the specimen. Organized as for instance an RO-Crate, each such item unit is an FDO that researchers can refer to externally and make statements about within the KG.

The partonomy that describes the anatomical organization of the specimen is modelled using interconnected *has-part* statement units. The collection of these statement units is organized in the KG as an RO-Crate and thus an FDO that is a granularity tree unit. Again, researchers can refer to the partonomy externally and make statements about it within the KG.

Finally, the entire subgraph representing the article in the KG is organized as an RO-Crate that is a compound unit of the type scholarly article unit. As an FDO, researchers can again refer to it and make statements about it.

When modelling the scientific findings of several scholarly articles in such a CLEAR scholarly KG, the graph will be organized hierarchically into several scholarly article units that each comprises several item units and a granularity tree unit based on a particular collection of *has-part*, *has-shape*, *has-length*, *has-width*, and *has-positional-relationship-to* statement units. This would organize the graph into five distinct layers, from triples to statement units, compound units that are item and granularity tree units, compound units that are scholarly article units, and the graph as a whole (Fig. 7).

### Semantic units enhance data access and data exploration

FAIR and CLEAR KGs introduce novel data access and semantically meaningful exploration points, supporting *known-item search* and *browse* user needs, improving the way users interact with and extract information from KGs. We outline four key data access and exploration points facilitated by CLEAR KGs (see *Challenge 3*):

#### 1. Exploring by ontology class

KGs usually organize class-terms into a taxonomy, enabling users to browse this taxonomy and trigger predefined Cypher or SPARQL queries by selecting terms from it. This feature allows users to identify all instances of a selected term within the graph—like a sophisticated **keyword search**. In CLEAR KGs, such a keyword search would return a list of different types of FDOs, with each item in the list indicating the number of FDO instances that reference the respective class-term. Users can then decide which levels of representational and other types of granularities they are interested in, enabling **zooming in**, **filtering out**, and providing **detail-on-demand** in a more structured and semantically meaningful way than conventional KGs do. **Each semantic unit provides a defined view on a specific part of the KG**, providing users with more options for data exploration.

Additionally, since every semantic unit instantiates a corresponding semantic unit class, one could also search and explore the KG using a **dynamic facetted search**, allowing users to filter search results using semantically relevant facets like FDO classes, in the case of statement units filtered by resource types and value ranges and patterns for each slot of the corresponding SHACL shape, and for nested FDOs by a list of associated statement FDO classes.

By enriching the taxonomy of class-terms with statistics about the number of their instances and the number of their mentions in statement, item, item group, granularity tree, and all other types of FDOs (for more types of statement and compound units, see discussion in [73]), coupled with the possibility to choose between different time intervals of their creation or last-updated date (e.g., within last week, last month, last year, last decade), FDOs that correspond with semantic units support the identification of hot-spots within CLEAR KGs at various levels of granularity, providing users with additional overview options.

#### 2. Exploring by semantic unit class

Each FDO in a CLEAR KG is represented by its own GUPRI and instantiates a corresponding FDO class. The set of all FDO classes forms a taxonomy, a nested hierarchy, with specialized FDO types being subclasses of more general parent FDO types. This taxonomy of different FDO types and their corresponding semantic units provides a **basic classification** for the contents of CLEAR KGs, with each individual FDO instantiating at least one corresponding FDO/semantic unit class. Users can access data by browsing the list of these classes.

The ability to differentiate FDO classes based on subject and object resources, value ranges, and in the case of nested FDOs their types of associated statement and other nested FDOs, allows users to identify the specific types of FDOs they seek. Predefined Cypher and SPARQL queries can then retrieve the instances of respective semantic unit classes, and users can access the information at the level of representation, the granularity perspective, and the frame of reference they are interested in and filter it according to their preferences using dynamic facets. The different types of FDOs can also be used for filtering the contents of a KG, simplifying the KG’s complexity by hiding irrelevant or highlighting relevant information.

Combining different types of FDOs and their corresponding semantic units in a search enables users to perform more comprehensive searches and access data at multiple levels. Users can, for instance, search for all instances of a specific type of statement FDO that are part of item FDOs that have a specific type of subject resource—e.g., all weight measurement statement FDO in phenotype description item group FDOs. Users can start with searching for instances of a specific ontology class, and after having selected a particular instance, the KG application can indicate which statements, items, item groups, granularity trees, datasets, questions, or which frames of reference this resource is part of. This assists users in **identifying relevant contexts**, indicating semantically meaningful opportunities to further explore the KG from a given data point. A simple click can start a predefined Cypher or SPARQL query, and the content-graph of a particular granularity tree or frame of reference that contains the previously selected resource can be shown in the user interface.

FDOs that correspond with semantic units can also be utilized for offering a summary of the contents of CLEAR KGs at different degrees of generalization and abstraction by indicating the number of instances of each FDO class. This summary aids in **profiling a KG** and gaining a general overview of its content. The human-readable descriptions associated with each statement FDO class in a CLEAR KG offer further valuable guidance for users exploring the KG. These descriptions provide insights into the type of information covered by a given type of FDO and can be displayed as tooltip texts in a user interface, enhancing the user experience whenever the respective class or one of its instances is presented.

Implementing the concept of semantic units as FDOs in KGs will thus contribute to a solution to all three challenges, that of overly complex machine-actionable graphs (*Challenge 1*), the challenge that many users and even software developers do not know query languages and do not want to learn them (*Challenge 2*), and the challenge of supporting contextual exploration and visual information seeking strategies in KGs (*Challenge 3*).

#### 3. External access through GUPRI

The GUPRI associated with each resource in a CLEAR KG allows external applications to refer to these resources unambiguously. Providing corresponding APIs enables external access to the CLEAR KG’s resources, including instances, classes, and semantic units. As FDOs, semantic units can also offer provenance data and contextual metadata, facilitating data integration with external sources.

#### 4. Tabular presentation

Statement units of the same type hold information of the same type, modeled according to the same shape. Therefore, their data are comparable and interoperable, making it feasible to present and visualize them in a single table, with each slot of the corresponding SHACL shape defining a column in the table and each statement unit instance taking a row in the table.

Item units with the same type of subject resource tend to have similar associated statement units. By aligning statement units across item units, a single table can visualize the contents of multiple item units or item group units, with statement units being mapped to the rows and the item units to the columns. The results of queries can be visualized using a similar tabular approach.

### Semantic units enable novel user interfaces for graph exploration in CLEAR knowledge graphs

CLEAR KGs present a promising framework for developing innovative user interfaces that support efficient **navigation** and **exploration**, aligning with the **visual information seeking strategies** of *‘Overview first’*, *‘Search first’*, and *‘Details first’* (*Challenge 3*).

**Fig. 8:**
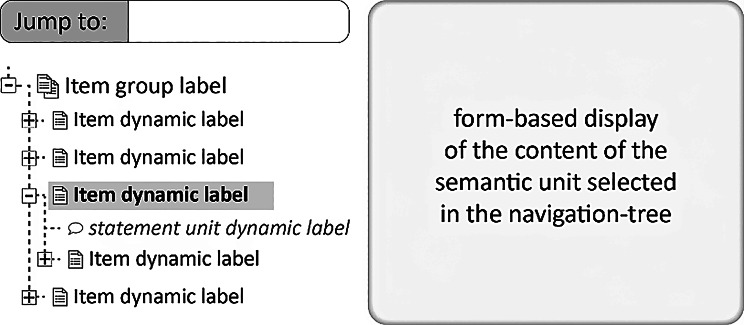
Navigation tree for navigating and exploring data of a specific item group unit. The tree to the left represents the hierarchy of a specific item group unit with all its associated item units, with ‘child’ item units describing in more detail a resource that their ‘parent’ item unit referred to as an object of one of its statement units. Users can use this tree for navigating the contents of the item group unit, zooming in and filtering out information, and getting more details on demand. The information from the selected item unit will then be displayed in a form, e.g., in a widget to the right. This way, users can have the items they are interested in expanded within the navigation tree and can easily jump between them by a simple click. Each semantic unit can be shown with its dynamic label

Apart from offering diverse access points for search and exploration (see above), user interfaces can incorporate a navigation tree for seamless browsing of contents related to a specific item group FDO or granularity tree FDO. This navigation tree builds on the hierarchy of interconnected item FDOs within the same item group FDO, which can become rather deep, with chains of multiple item FDOs connected via statement FDOs. By representing this navigation tree in the user interface in a folder-like system, utilizing the human-readable dynamic labels of the corresponding semantic units, users can intuitively jump from one item FDO to another by a single click (Fig. 8). Since item FDOs can be linked to each other via different types of statement FDOs, one can even allow users to filter for specific types of linking statement FDOs. Consequently, a user can for instance decide that they only want to see item FDOs that are linked to each other via adjacency statement FDOs and the navigation tree would show the respective item FDO tree(s).

**Fig. 9:**
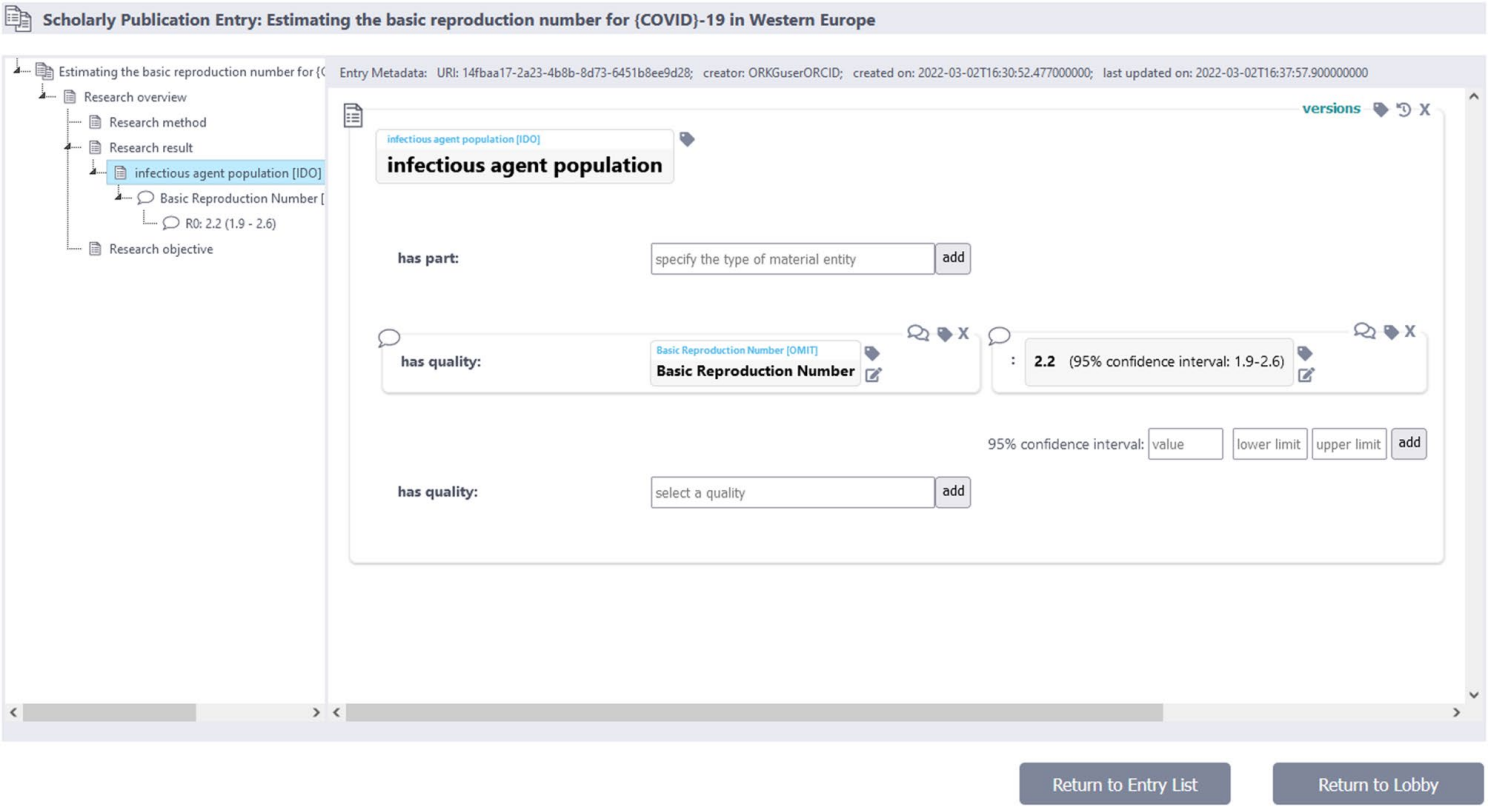
User interface showing a scholarly publication item group unit. The left widget shows a navigation tree with the item group unit at the root (i.e., top) and all associated interlinked item units organized below in the tree. The content of the selected item unit is displayed in the widget to the right, using Jinja templates. The item unit describing ‘infectious agent population’ (IDO:0000513) is selected, and it has two associated statement units that specify the population’s basic reproduction number. Input fields allow adding more data

To demonstrate this navigation tree functionality, we have developed a corresponding user interface in a Python-based prototype of a FAIR and CLEAR scholarly KG application [96] (Fig. 9). The prototype exemplifies the core concept, though it lacks the ability to select different types of linking statement units. Additionally, the prototype includes versioning for semantic units, automated tracking of editing histories and provenance information. It is available from https://github.com/LarsVogt/Knowledge-Graph-Building-Blocks.

**Fig. 10:**
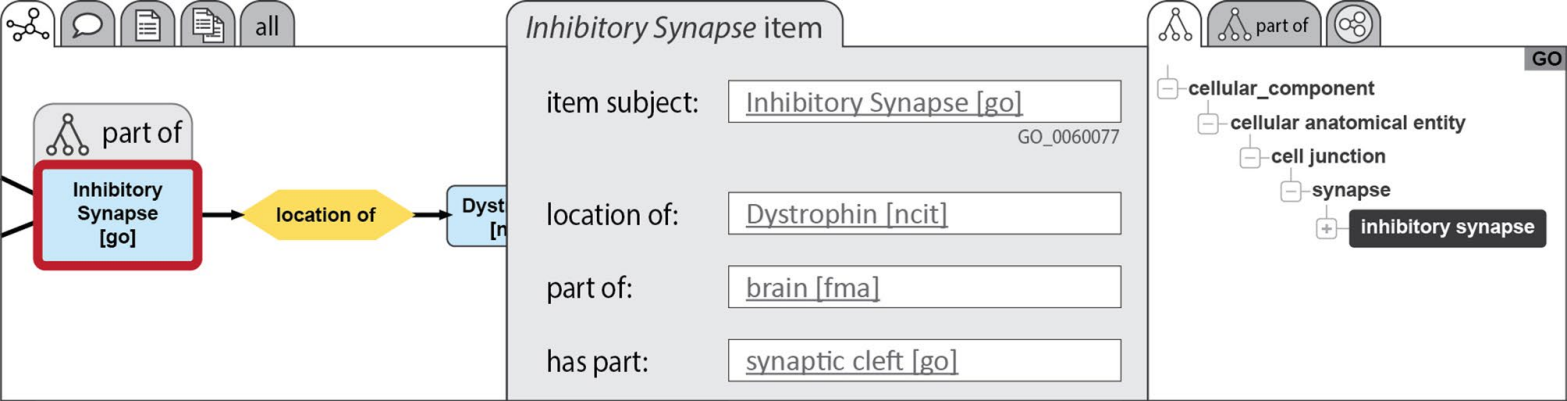
Concept study of a user interface with interacting mind-map like graphical and form-based textual widgets for knowledge graph exploration. The user interface is divided into three parts: to the left, the dynamic mind-map graphical exploration widget for zooming in and out across all five levels of representational granularity, indicated by the tabs at the top left (triples, statements, items, item groups, the whole graph). Currently, the triples level is shown, and the ‘inhibitory synapse’ (GO:0060077) node is selected. This graphical user interface allows users to explore the graph, select a node or a relation and, by clicking on one of the tabs above, switch to their representations at different levels of representational granularity. The form-based textual widget in the middle shows the input form for the *’inhibitory synapse item unit’* and allows users to change, delete, or add new statement FAIR Digital Objects (FDOs), with the other two widgets being updated automatically. To the right, the graphical representation of the taxonomy for the currently selected resource is displayed, with tabs allowing users to switch to other types of granularity trees (e.g., a parthood granularity tree) or frames of reference. When a user selects a relation in the left widget, the middle widget will show the corresponding statement FDO with its associated provenance information and the right widget the taxonomy of statement FDOs. Clicking into an input field in the form widget in the middle will select the corresponding subgraph in the graph widget to the left. All dynamic mind-map displays in the graph widget to the left do not show the triples as they are stored in the machine-actionable content-graph but utilize the dynamic mind-map patterns that only consider the information that is relevant to a human user, guaranteeing the human-actionability of the data and metadatacontent-graph

Often, **graphical representations** in the form of mind-maps are better suited for understanding relationships between entities of interest than textual representations [Criterion 3]. However, conventional KGs often suffer from information overload, typically containing a lot of information that is only relevant to a machine, rendering the graph unnecessarily complex and less comprehensible for human readers (see, e.g., Fig. 1 Middle; *Challenge 1*). With the introduction of dynamic mind-maps and the distinction of representational granularity levels, CLEAR KGs allow for filtering the data graph into semantically meaningful subgraphs. The dynamic mind-maps present only information that is relevant to users, reducing the complexity of the underlying data graph to easily digestible chunks for a human reader. Users can zoom in and out across levels, traverse granularity trees, and explore different frames of reference in a manner that aligns with their interests. Users can, for instance, start to explore the graph with a search result that shows an item group FDO that contains information they searched for and all relations of this FDO to other item group FDOs (e.g., an observation-statement from publication *A* supports a hypothesis-statement in publication *B*). Moreover, all associated statement FDOs of a given item FDO could be shown and thus information from the next finer representational granularity level (Fig. 10).

The identification of hierarchical structures like granularity trees and frames of reference via context FDOs empowers user interfaces of CLEAR KGs to facilitate **smart and semantically meaningful exploration**. By displaying **relevant contexts**, users can effortlessly expand the graph in the direction of their interests. The graph-exploration tools of user interfaces of CLEAR KGs could support this process by allowing users to select specific nodes and explore the graph accordingly. Indications of associated types of statement and compound units aid in filtering the displayed dynamic mind-map representations of statement and nested FDOs, streamlining the user’s exploration journey (Fig. 10).

These exploratory tools and visualizations can be combined in a seamless and intuitive manner, enabling users to switch between text-based, form-based, and graphical presentations of the data, providing users with a holistic and versatile approach to engaging with the information contained within a CLEAR KG (Fig. 10).

## Conclusion

In this paper, we argue that two prerequisites must be distinguished to be able to answer the question of what makes data and metadata FAIR and actionable for humans and machines alike:

1. **Structural FAIRness of data and metadata**: There are specific structural prerequisites that are required for data and metadata to be FAIR and actionable, which include.
  a. **technical properties**: Data and metadata must be structured in specific ways to be FAIR and actionable, including their format, underlying schema, and the vocabulary used, and these structural elements must be combined into an ideally broadly accepted standard. The EOSC Interoperability Framework addresses this aspect of FAIRness.
  b. **cognitive properties**: Data and metadata must be structured in such a way that humans can easily comprehend the meaning they carry. Adding cognitive interoperability as a fifth interoperability layer to the EOSC Interoperability Framework would address this aspect of FAIRness.
2. **Operational FAIRness of data and metadata**: Considering the large amounts of data that users often face when tackling a research question, users need tools supporting their data exploration, searching, and processing tasks. These task-specific tools must.
  a. **support different exploration strategies**: Visual information seeking strategies such as *‘Overview first’*, *‘Search first’*, and *‘Details first’*, which also require some structural properties of data and metadata.
  b. **be usable**: Users must be able to find the tools and use them properly without too much effort involved. This aspect of FAIRness is also addressed by the cognitive interoperability of data and metadata.

With its focus on cognitive interoperability, we can infer from operational FAIRness additional structural requirements for FAIR data and metadata (and corresponding KGs), leading to the proposal of the **CLEAR Principle** of data and metadata that complements the FAIR Guiding Principles. While we introduced and discussed the CLEAR Principle in reference to KGs, its underlying main principles are independent of this technology and can be applied to other information management systems by partitioning their data layer into statement FDOs and by defining semantically meaningful collections of statements as nested FDOs.

The overall goal of adding cognitive interoperability to the EOSC Interoperability Framework and proposing the CLEAR Principle is to address the key challenge of striking a balance between machine-actionability and human-actionability in information technology systems to enable efficient data management and exploration. We argue that reaching this goal requires understanding FAIRness not solely as a structural property of data and metadata, but also as depending on a comprehensive FAIR Services ecosystem consisting of a terminology, a schema, and an operations service [65].

While the FAIR Guiding Principles focus on findability, accessibility, interoperability, and reusability of data and metadata, they do not explicitly emphasize cognitive interoperability or the human contextual explorability of data and metadata. In contrast to the FAIR Guiding Principles, the CLEAR Principle provides guidance regarding:

1. **Cognitive interoperability**: data and metadata structures must be aligned with cognitive processes, enabling intuitive interaction, interpretation, and understanding for human users of CLEAR KGs;
2. **semantically Linked** **data and metadata**: data and metadata must be structured and organized into semantically meaningful units of information to provide meaningful context for human users;
3. **contextually** **Explorable data and metadata**: it must be possible for a user of a CLEAR KG to seamlessly navigate, query, and contextually explore the graph, thereby keeping the cognitive overload for its users at a minimum;
4. **intuitively Accessible data and metadata**: accessibility of data and metadata in a CLEAR KG must be well aligned with human cognition;
5. **human-Readable and -interpretable data and metadata**: data and metadata must be presented in a way that is easily comprehensible for users of a CLEAR KG (human-actionability), while still providing data and metadata structures that support their machine-actionability.

The CLEAR Principle complements the FAIR, CARE, and TRUST principles by addressing the human-centric aspects of data and metadata interoperability―something largely missing in current frameworks. Cognitive interoperability and human explorability of data and metadata are aspects of data management that attracted little attention in the context of KGs. With semantic units organized as FDOs, we introduce a concept that provides a framework that supports cognitive interoperability and that meets the specifications of the CLEAR Principle. Semantic units add resources to CLEAR KGs that are of a higher level of abstraction than the low-level abstraction of resources used in the triples of conventional KGs. Technically, semantic unit resources and their corresponding FDOs instantiate corresponding FDO classes, while semantically, they represent statements or interconnected sets of semantically and ontologically related statements that are represented as semantically meaningful units of information. This organizational structure not only partitions the content-graph layer of a CLEAR KG into different levels of representational granularity, granularity perspectives, and frames of reference, but also introduces a comprehensive framework for making statements about statements.

Within CLEAR KGs, semantic units play a pivotal role in enhancing the FAIRness of data and metadata, because every semantically meaningful proposition is organized as a separate statement FDO that instantiates a corresponding statement FDO class. By referencing a data schema and corresponding query patterns in the statement FDO class for create, read, update, and delete (CRUD) tasks, each statement FDO references via its class affiliation the underlying data schema. Because every triple of the content-graph layer belongs to exactly one statement FDO, all data in CLEAR KGs have information about their underlying data schema and how to query them, thus contributing to the overall FAIRness of their data and metadata. This inherent structure not only aids users in finding specific data points of interest but also empowers developers with CRUD query patterns, all while **circumventing the need for developers and users to learn graph query languages** (*Challenge 2*).

Our proposed user interface framework builds upon semantic units, offering novel ways of navigating and exploring CLEAR KGs, filtering for information of interest, and providing multiple entry-points for accessing and exploring the graph, zooming in and out across different levels of representational granularity, highlighting and making accessible any granularity trees and frames of reference in the data. Semantic units also contribute to **resolving the conflict and dilemma between machine-actionability and human-actionability of data and metadata** in a CLEAR KG (*Challenge 1*), since data representations can provide the contextual information required for machines, while humans can access and explore a graph of reduced complexity by utilizing graph-based displays based on dynamic mind-maps or form-based textual displays based on dynamic labels. Consequently, semantic units provide the framework required for user interfaces to align with the visual information seeking strategies of *‘Overview first’*, *‘Search first’*, and *‘Details first’*, providing additional structure that supports the development of new workflows and accompanying user interfaces for **KG exploration approaches such as KG profiling and summarization**,** KG exploratory search**,** and KG exploratory analytics** (*Challenge 3*).

By organizing statement units as nanopublications, forming statement FDOs, and by organizing compound units as RO-Crates, forming nested FDOs, semantic units with their taxonomy of semantic unit classes provide a promising framework for implementing different types of FDOs, lending itself to federated nested FDOs where the actual data in the form of statement FDOs can be distributed across different stakeholders, possibly supporting explorability of data and metadata over a federated KG.

In summary, semantic units lay the foundation for cognitive interoperability in CLEAR KGs, substantially increasing their terminological and propositional interoperability. With their ability to decouple data display from storage, they provide a powerful solution for presenting complex data to human users while ensuring machine-actionability. This innovation fosters a collaborative environment for sharing data among stakeholders, while data stewardship remains with domain experts or institutions (following Barend Mons’ *data visiting* as opposed to *data sharing* [97]). The modular and nested nature of FDOs based on semantic units promotes broader accessibility and adoption for both software developers and users who lack expertise in semantics, thus fostering the wider FAIRification of data and metadata.

As we move forward, we envision a future in which CEAR KGs function as dynamic and intuitive knowledge repositories, providing seamless support for both human exploration and machine interaction. This will lead to significant advancements in the field of data management and knowledge representation. The analogy here is that of a lake with clear water, which allows humans standing at the shore to see to the bottom of the lake and observe all fish swimming in it. Similarly, CLEAR KGs enable their users to effortlessly find and comprehend all data and metadata relevant to them within the KG.

## Data Availability

No datasets were generated or analysed during the current study.

## Footnotes

1 The CARE Principles for Indigenous Data Governance: Collective benefit, Authority to control, Responsibility, and Ethics. The CARE Principles have been developed in consultation with Indigenous Peoples, scholars, non-profit organizations, and governments to reflect the important role of data in advancing innovation, governance, and self-determination among Indigenous Peoples [24].

2 A metonymy is the substitution of the name of an attribute or adjunct for that of the thing meant, for example, *“the pen is mightier than the sword”* with *“the pen”* referring to writing and *“the sword”* to warfare and violence.

3 Here, the focus is on the communication of semantic conceptual content and thus content organized and documented in the form of texts and symbols. Considering the communication of perception-based non-conceptual content, as when using media such as images, diagrams, audios, and videos, is out of the scope of this paper.

4 A nanopublication is defined as a diminutive KG snippet accompanied by metadata, which is regarded as an autonomous scientific publication. The content of a nanopublication may encompass a wide range of subjects. Nanopublications are articulated in a KG format that is both formal and machine-interpretable. The dissemination of individual data as independent publications, accompanied by either a research article or not, is facilitated by nanopublications. A distinguished feature of nanopublications is their capacity for attribution and citation, which serves as an incentive for researchers to make their data available as nanopublications, thereby promoting data accessibility and interoperability.

5 An RO-Crate is defined as a Research Object (RO) comprising a collection of data (Crate) and designated RO-Crate-metadata.json file that provides a description of the collection. The nature of the collection is versatile, encompassing any type of research data―including but not limited to papers, data files, software, references to other research, and other pertinent materials. The collection may manifest in various forms and could take the form of a collection of nanopublications.

## Abbreviations

BFO: Basic Formal Ontology
CLEAR: Cognitively interoperable, semantically Linked, contextually Explorable, intuitively Accessible, and human-Readable and -interpretable
CLEAR KG: CLEAR Knowledge Graph
EOSC: European Open Science Cloud
FAIR: Findable, Accessible, Interoperable, and Reusable
FDO: FAIR Digital Object
FMA: Foundational Model of Anatomy Ontology
GDPR: General Data Protection Regulation
GO: Gene Ontology
GUPRI: Globally Unique Persistent and Resolvable Identifier
IAO: Information Artifact Ontology
ID: Identifier
IDO: Infectious Disease Ontology
KG: Knowledge Graph
LLM: Large Language Model
NCI: National Cancer Institute
NCIT: NCI Thesaurus OBO Edition
NoSQL: Not only SQL
OBI: Ontology for Biomedical Investigations
OBO: Open Biological and Biomedical Ontology
OMIT: Ontology for MIRNA target
OWL: Web Ontology Language
PATO: Phenotype and Trait Ontology
RDF: Resource Description Framework
RO OBO: Relations Ontology
RO-Crates: Research Object Crate
SEMUNIT: Semantic Unit Ontology (currently in development)
SHACL: Shape Constraint Language
SPARQL: SPARQL Protocol and RDF Query Language
SQL: Structured Query Language
STATO: The statistical methods ontology
UBERON: Uber-anatomy ontology
UO: Units of Measurement Ontology
